# A study of unreasonable illegitimate tasks, administrative tasks, and sickness presenteeism amongst Norwegian physicians: an everyday struggle?

**DOI:** 10.1186/s12913-018-3229-0

**Published:** 2018-06-05

**Authors:** Sylvi Thun, Vidar Halsteinli, Lise Løvseth

**Affiliations:** 1 Department of Research and Development, Division of Psychiatry, St. Olavs Trondheim University Hospital, Trondheim, Norway; 2 Regional Centre for Health Care Development, St Olavs Trondheim University Hospital, Trondheim, Norway

**Keywords:** Administrative tasks, Exhaustion, Physicians, Role conflict, Sickness Presenteeism, Unreasonable illegitimate tasks

## Abstract

**Background:**

It has been shown that a recently defined stressor, ‘illegitimate tasks’, has negative effects on employees’ work motivation and health. Better understanding of the illegitimate tasks undertaken by physicians might contribute to a more resource-efficient division of labour within the health care system, with beneficial effects on organisational economics and employee performance. We aimed to investigate the prevalence of unreasonable illegitimate tasks, their associations with workplace variables and their impact on health, in particular sickness presenteeism.

**Methods:**

Cross-sectional data were collected in 2012. A sample of 545 Norwegian physicians answered an online questionnaire. The response rate was high (71.8%). The data were analysed using independent-samples *t*-tests, ANOVA and logistic regression.

**Results:**

About 50.2% of physicians in all clinical positions reported that at least 11% of their everyday tasks could have been done by other hospital personnel. Seven percent of the physicians reported that at least 31% of their daily workload consisted of unreasonable illegitimate tasks. There were no significant differences in unreasonable illegitimate tasks according to clinical position, age or gender. Administrative task load and role conflict were positively associated with unreasonable illegitimate tasks that physicians reported could be reallocated to non-medical professionals. Moreover, unreasonable illegitimate task was associated with a higher probability of sickness presenteeism after controlling for age, gender, role conflict, control over work pace, exhaustion and administrative tasks.

**Conclusions:**

The results confirm that physicians’ workload includes a high proportion of unreasonable illegitimate tasks and that this can contribute to sickness presenteeism. Investigation of work environmental factors can provide insight into the mechanisms behind unreasonable illegitimate tasks. Decreasing the amount of administrative tasks and role conflicts faced by physicians should be a priority. These findings could be used to make hospital task management more resource-efficient. Our results indicate that a substantial proportion of physicians’ work capacity could be re-allocated to core tasks. Further research is needed into the specific type and content of unreasonable illegitimate tasks undertaken by physicians in order to determine to whom they should be allocated to ensure a healthy and motivated workforce and provision of high quality, resource-efficient health care services.

## Background

It has been shown that a recently defined stressor related to improper use of work time, termed ‘illegitimate tasks’ [1, 2], is related to stress and well-being at work [3–7]. Findings from both Europe and the US have been published [1, 4, 8, 9], but findings on illegitimate tasks and their relationship with stress outcomes need to be validated in samples from different cultures and occupational groups [1, 2, 10]. In addition, because organisations and employees aim to minimise the frequency of illegitimate tasks, there is a need to explore possible antecedents of illegitimate tasks [4]. Efficiency has for many years been a key policy goal in many Western countries [11] and increased knowledge about the extent and content of the illegitimate tasks undertaken by physicians would help to improve the efficiency of health services. There is evidence that there has been an increase in the proportion of work time that physicians spend on non-core tasks [12], raising concern that physicians’ time is not being used appropriately. This inappropriate use of physicians’ work time can be associated with performance of illegitimate tasks. To our knowledge there has not yet been any systematic investigation of the proportion of physicians’ workload that is made up of unreasonable illegitimate tasks. The aim of this study was to investigate the prevalence of unreasonable illegitimate tasks, associated psychosocial work environment factors and one health-related outcome amongst physicians working in a hospital setting.

### Theoretical background

Illegitimate tasks, defined as work tasks that are not related to one’s core occupation or that one perceives as inappropriate and a waste of time, are an emerging issue in occupational stress research [2, 7]. If an employee perceives that a work task should have been carried it out by others, or if it violates norms about expected work tasks or challenges his or her professional identity then it is considered illegitimate [1, 2]. The concept of illegitimate tasks is derived from the stress-as-offense-to-self (SOS) theoretical framework, which posits that being assigned an illegitimate task may trigger stress reactions [1], based on Lazarus’s [13] assumption that stress is related to threats to important goals. There are two types of illegitimate tasks: unnecessary and unreasonable [1, 2, 7]. Unnecessary illegitimate tasks are defined as tasks that are perceived as a waste of time and need not be done by anyone, and tasks that could have been avoided or carried out with less effort if things were organised more efficiently [2], e.g. transferring patient data manually because two technological systems are incompatible. Unreasonable illegitimate tasks are tasks that are outside one’s occupational role and conflict with specific aspects of one’s role or occupational status [2], e.g. physicians asked to transporting a bedbound patient are likely to criticise the organisation for taking them away from their core task, which is medical treatment, whereas a hospital orderly would see the same task as part of his or her daily work [4]. At the theoretical level it has been argued that unreasonable and unnecessary illegitimate tasks should be treated as separate constructs [8]. In this study we investigate reports of unreasonable illegitimate tasks. Unreasonable illegitimate tasks are considered the main facet of illegitimate task and deserve research attention because of their association with work engagement and time pressure [14].

Physicians can be described as cost-generating professionals because they have high salaries and demand additional treatment resources [15], so it is inefficient to have physicians carrying out tasks that could be performed by others and it has been suggested that research into the medical profession’s experience of illegitimate tasks is needed [3]. Physicians’ perception of illegitimate tasks can depend on a variety of factors within their work context [16]. Ahmned, Eatough, and Ford [17] found national variations in illegitimate task burden and outcomes of undertaking illegitimate tasks. This study investigated physicians at a Norwegian university hospital. As part of the public health care system, hospitals are owned by the government, run by regional health authorities and publicly funded as part of the national budget. Part of hospitals’ income is activity-based, but physicians are permanent employees with fixed salaries. General practitioners are self-employed, but publicly financed, partly on a fee-for-service basis. In addition Norway has a strong Work Environment Act and strong employment protection legislation. There is also a relatively low power distance between employees and employers in Norway [18]. Power distance is the degree to which less powerful members of a society are comfortable with the unequal distribution of power and recognise it as legitimate [18]. In Norway, as in many other countries, there has been a change in task management and an increase in the amount of work time spent by employees on additional tasks and administration. Accordingly, it is possible that illegitimate tasks are experienced as a threat to time spent on core tasks.

Health care workers, including physicians, report spending increased amounts of time on meetings, administration, measurement and reporting rather than working with patients [3, 12, 19]. The growing number of administrative tasks diverts time and attention away from more clinically important activities [19]. These administrative tasks are not unnecessary, but are likely to fall - to some extent - outside of the range of tasks physicians consider part of their professional role. Hence there is a need to explore how administrative burden relates to reports of illegitimate tasks. A better understanding of illegitimate tasks should make it possible to make better decisions about task assignment [7] and task management. Ahmed et al. [17] highlighted the risk that as a result of increased focus on the economic growth, employees will find themselves assigned more tasks, including administrative tasks, that fall outside their formal job role. More knowledge of the correlates of illegitimate tasks should enable organisations to reduce their number [10] or find ways to manage them in a more resource-efficient manner.

As illegitimate tasks have been shown to act as a job stressor it is important to investigate how they affect physicians’ health and well-being [4, 7, 20]. Previous research has shown that illegitimate tasks are negatively related to job satisfaction [4, 6] and positively related to counterproductive behaviour [2] and stress reactions among employees [21]. One study found that participants who experience a high burden of illegitimate tasks had higher levels of the stress hormone cortisol than peers dealing with fewer illegitimate tasks [22]. In addition, illegitimate tasks have been shown to reduce sleep quality [23], and have been prospectively associated with lower mental health [5] as well as being correlated with negative affect and low self-esteem at the end of the working day [8]. Illegitimate tasks have also been found to be strongly associated with strain outcomes, such as feelings of resentment towards one’s organisation and irritability, over a period of 2 months [7], and have been related to turnover intention [10]. Overall, previous research indicates that illegitimate tasks have negative consequences for both individuals and organisations, but none of these studies has looked at the experiences of physicians. We argue that in order to improve our understanding of unreasonable illegitimate tasks it is important to study the phenomenon in a specific profession, as illegitimate tasks are a specific, task-related stressor that constitutes a threat to one’s professional identity [7, 9].

The SOS theoretical framework implies that being expected to undertake illegitimate tasks can increase the risk of poor health amongst physicians. Performing illegitimate tasks requires emotional and physical effort and may induce symptoms of strain [7]. Cognitive, affective and physical strain may result in exhaustion [24], and exhaustion has been shown to correlate with illegitimate tasks [3, 7, 25]. Exhaustion has also been shown to have a reciprocal relationship with sickness presenteeism [26], defined as attending work when ill [27]. Amongst physicians strain and ill health often manifest as sickness presenteeism [28–30], which has been shown to be prevalent among physicians [28, 30, 31]. Physicians often go into work when they are ill, because of their high work load, the lack of a replacement, responsibility and their crucial role in hospitals’ main task, which is medical treatment [28, 30, 32]. Illegitimate tasks can be regarded as stressors that can contribute to strain and a variety of negative health outcomes [21]. The job demands-resources model [33, 34] provides a theoretical framework which explains why unreasonable illegitimate tasks may be associated with sickness presenteeism. Unreasonable illegitimate tasks may be considered job demands, as it requires effort to carry them out and they impose demands on individuals who do so [7]. It is possible that in a bid to cope with the pressure of unreasonable illegitimate tasks, physicians use sickness presence as a strategy for coping with their high workload and so the relationship between unreasonable illegitimate tasks and sickness presenteeism should be investigated [3].

A high prevalence of tasks perceived as illegitimate can imply that an organisation would benefit from allocating resources differently. As previous research on illegitimate tasks has reported that they have consequences for both individuals and organisations, there is a need for studies of the prevalence of illegitimate tasks in different professions [2, 4, 7]. Although there have been advances in research on illegitimate tasks studies of the relationships between illegitimate tasks, workplace correlates and health-related outcomes are scarce [4, 9]. A job characteristic associated with illegitimate tasks, especially unreasonable illegitimate tasks, is role conflict, defined as lack of congruent expectations and demands from other people in the workplace [35, 36]. As the concepts of role conflict and illegitimate tasks are highly related but distinguishable from each other, it is important to control for role conflict in research on illegitimate tasks [1, 2, 7]. In addition, performance of work tasks that are outside their core role or job definition has been associated with stress and ill health in physicians [19, 37]. Investigating the relationship between unreasonable illegitimate tasks and health outcomes is important, as physicians’ ill health can indirectly or directly affect the quality of health care and patient safety [38–40].

Based on the theoretical framework and review of previous findings, the aim of this study was threefold: 1. To investigate what proportion of working time hospital physicians think they spend on unreasonable illegitimate tasks and how this varies with clinical position, gender, age and reported administrative workload. 2. To analyse the relationship between unreasonable illegitimate tasks and reported administrative work amongst physicians whilst controlling for variance in role conflict, gender and age. 3. To describe the relationship between unreasonable illegitimate tasks and sickness presenteeism in physicians after controlling for variance in age, gender, role conflict, control over work pace, exhaustion and administrative tasks.

## Method

### Participants and procedure

The study is based on survey data from a sample of Norwegian university hospital physicians participating in a study concerning work-related health, organisational culture and working conditions amongst European physicians (HOUPE study, phase II). We conducted a web-based survey, in English, between February and May 2012. Participants received a letter describing the study and containing a link to the questionnaire; they provided their responses anonymously. The participants were qualified physicians and in full- or part-time employment at the time of data collection. The project was approved by the hospital board, the union representatives of the physicians at the hospital and the Regional Ethics Board. As well as sending out an email giving information about the project, the research team also gave short, oral presentations to all departments and clinics at appropriate meetings. Participation was voluntary and the online survey could not be accessed until consent to participation had been provided. The response rate was 71.8% (*N* = 545/759). Just under half the participants (45%, *n* = 245) were female physicians. An analysis of non-respondents showed that the sample was representative of all physicians of the hospital in terms of age, gender, and position.

### Measures

The questionnaire included 123 items on education, work-related health, organisational culture and working conditions. Here we report on the variables relevant to our objectives: illegitimate tasks, role conflict, control over work pace, amount of administrative tasks, exhaustion and sickness presenteeism.

#### Unreasonable illegitimate tasks

The proportion of physicians’ workload made up of unreasonable illegitimate tasks was measured by the item “In your opinion, what proportion of the tasks you deal with every day could be done by other hospital personnel (non-physicians)?” The response options were: none (coded 1); 5–10% (2); 11–20% (3); 21–30% (4); ≥31% (5). The data from the ordinal variable were recoded into a binary variable: low level of unreasonable illegitimate tasks (0 = 0–10%) and high level of unreasonable illegitimate tasks (1 = ≥11%) to enable analysis of mean differences in work environment factors on the basis of level of unreasonable illegitimate tasks. The cut-off between low and high level of unreasonable illegitimate tasks was based on Eatough et al.’s [9] findings that illegitimate tasks can occur several times within a workday, with an average of 2 and 3 such tasks per week.

#### Personal characteristics

Participants were asked to describe their current clinical position using one of the following categories: resident doing specialist training (1), specialist/consultant/medical officer (2), chief physician/senior medicalofficer/director (3) or other (4). Gender was coded with male (1) as the reference category. Age was captured using nine age categories: ≤ 29 years (1); 30–34 years (2); 35–39 years (3); 40–44 years (4); 45–49 years (5); 50–54 years (6); 55–59 years (7); 60–64 years (8) ≥ 65 years (9).

#### Administrative/management tasks

The respondents were asked to report the percentage of their work time that was taken up by management and administration tasks.

#### Role conflict

Role conflict was measured using three items from the General Nordic Questionnaire for Psychological and Social Factors at Work (QPS Nordic) [41]. The selected items deal with conflicts between demands and resources, conflicting requests and conflicts between the subject’s expectations and external demands [42]. Example item: “How often do you receive incompatible requests from two or more people?” Responses were given on a five-point scale ranging from “very seldom or never” (1) to “very often or always” (5). Cronbach’s alpha for this scale (α = .68) corresponded to the validation data on QPS Nordic [41, 42]. A high score indicates high role conflict.

#### Control over work pace

We measured control over work pace with four items from the QPS Nordic [42] dealing with control over work pace, decisions about the timing and length of breaks and decisions about working hours (flexitime). Responses were given on a five-point scale ranging from “very seldom or never” (1) to “very often or always” (5). Cronbach’s alpha for the scale (α = .83) corresponded to the validation data on QPS Nordic.

#### Exhaustion

Exhaustion was measured using five items from the Oldenburg Burnout Inventory (OLBI) [24] (α = .73). The measure included both positively and negatively worded items (revised). Example item: “After work I usually feel worn out and weary”. Responses were given using a four-point Likert scale ranging from “totally agree” (1) to “totally disagree” (4). High scores on the burnout measures indicate a feeling of exhaustion.

#### Sickness presenteeism

This was measured with two questions: “Have you gone to work with an illness in a situation where you would have recommended a patient to stay at home?” followed up by “How often has this happened during the last 12 months?” [29]. Responses were coded as follows: none (1), once (2), 2–4 times (3) and > 5 times (4). Employees were classified as showing sickness presenteeism if they had attended work whilst ill as least twice during the previous 12 months [43]. The variable was dichotomised as regular attendance (0) and sickness presenteeism (1).

### Statistical analyses

The illegitimate tasks variable is reported as a percentage and frequency. One-way ANOVA was used to assess differences in illegitimate task load on the basis of clinical position. Independent samples *t*-tests (two-tailed) were used to assess mean differences between psychosocial strain variables in participants with low and high loads of illegitimate tasks. Logistic regression models were used to describe correlates of illegitimate tasks and illegitimate tasks as a correlate of sickness presenteeism. We confirmed that the data met the requirements for logistic regression analysis [44, 45]. SPSS version 24 was used for the analyses.

## Results

### Descriptive statistics

Table 1 presents means, standard deviations and correlations between the variables. The bivariate correlations indicated positive associations between unreasonable illegitimate tasks and role conflict (*r* = .20, *p* < 0.001), and sickness presenteeism (*r* = .12, *p* < 0.01). The variable most closely associated with unreasonable illegitimate tasks was administrative workload (*r* = .23, *p* < 0.001). The variable most associated with sickness presenteeism was exhaustion (*r* = .27, *p* < 0.001).

**Table 1:** Means (M), Standard Deviations (SD), and Correlations between the study variables

Measure	*M* (*SD*)	1	2	3	4	5	6
1. Unreasonable illegitimate Work Task	2.66 (1.08)	–					
2. Administrative Task	20.27 (18.14)	.23**	–				
3. Role Conflict	2.64 (0.80)	.20**	.06	–			
4. Control over Work Pace	2.76 (0.94)	.04	.15**	−.20**	–		
5. Exhaustion	2.48 (0.60)	.05	.04	.32**	−28**	–	
6. Sickness Presenteeism	2.47 (0.89)	.12*	.03	.17**	−24**	.27**	–

*p < .01 **p < .001

The question of whether unreasonable illegitimate tasks was prevalent amongst physicians was explored. Table 2 shows that 50.2% physicians reported that at least 11% of their everyday tasks could have been done by other hospital personnel. Approximately 7% of the physicians reported that at least 31% of their workload consisted of unreasonable illegitimate tasks. There were no mean differences of unreasonable illegitimate tasks according to clinical position, age or gender. Table 2 shows data on physicians’ unreasonable illegitimate tasks among all physicians and based on their current clinical position.

**Table 2:** Physicians’ unreasonable illegitimate task load as a percentage of task load (*n* = 538)

Unreasonable illegitimate Work Tasks	All Employees *n* (%)	Residents *n* (%)	Specialists *n* (%)	Others *n* (%)
None	69 (12.6)	28 (13.5)	41 (14.2)	2 (4.8)
5–10%	194 (35.5)	79 (38.2)	103 (35.6)	12 (28.6)
11–20%	166 (30.3)	65 (31.4)	85 (29.4)	16 (38.1)
21–30%	71 (13.0)	21 (10.1)	42 (14.5)	8 (19.0)
31% or more	38 (6.9)	16 (7.7)	18 (6.2)	5 (11.9)
Total *n*	538 (98.4)	207	289	42

We also analysed the associations between unreasonable illegitimate tasks and amount of administrative tasks after controlling for variance in role conflict, gender and age. The results from the logistic regression are summarised in Table 3. There was a positive relationship between unreasonable illegitimate tasks and amount of administrative tasks (*b* = .03, *p* < .001). In terms of interpreting the findings it is important to note that administrative tasks are coded in terms of percentage of working time meaning that the unstandardised coefficient reflects an increase of 1 %. Criteria set out by Cox and Snell and Nagelkerke [45] show that the logistic model explained between 10.4 and 13.9% of the variance in illegitimate tasks. These findings indicate that physicians who have a high administrative task load and experience conflict are also likely to report a high unreasonable illegitimate task load.

**Table 3:** Predictors of unreasonable illegitimate tasks amongst physicians

Variable	*b*	Wald	OR	95% CI for *OR*
Administrative Tasks	0.03*	21.41	1.03	[1.02–1.04]
Role Conflict	0.64*	25.40	1.77	[1.48–2.43]
Gender	−0.80	0.15	0.92	[0.63–1.36]
Age	−0.06	1.28	0.95	[0.86–1.04]
-2log likelihood 630.21* Cox & Snell’s *R*^*2*^ .104 Nagelkerke’s *R*^*2*^ .139				

*Note.* *p < .001

The analyses show that there were systematic differences in psychosocial variables based on unreasonable illegitimate task load. Participants with a high unreasonable illegitimate task load experienced greater role conflict (*M* = 2.82, *SD* = 0.75) than participants with a low unreasonable illegitimate task load (*M* = 2.44, *SD* = 0.80, *t* (534) = 5.62, *p* < 0.001). This was a medium effect (Cohen’s *d* = 0.49). Sickness presenteeism was greater in participants with a high unreasonable illegitimate task load (*M* = 2.61, *SD* = 0.85) than participants with a low unreasonable illegitimate task load (*M* = 2.34, *SD* = 0.91, *t* (535) = 3.57, *p* < 0.001). This was a medium effect (Cohen’s *d* = 0.31). These results show that a high unreasonable illegitimate task load is more stressful than a low unreasonable illegitimate task load.

Finally, we hypothesised that unreasonable illegitimate tasks would be related to sickness presenteeism after controlling for variance in age, gender, role conflict, control over work pace, amount of administrative tasks, and exhaustion. The results from the logistic regression are summarised in Table 4. They indicate that the unreasonable illegitimate tasks variable was a positive predictor of sickness presenteeism. The *OR* for unreasonable illegitimate tasks was 1.69 (*p* < 0.01), indicating that, on average, a physician who reports a high load of unreasonable illegitimate tasks had an *OR* for sickness presenteeism of 1.69. Exhaustion had the highest *OR* value (*OR* = 1.81, *p* < 0.001), indicating that as level of exhaustion increases the probability of attending work whilst sick also increases. The *OR* for control over work pace was 0.64 (*p* < 0.001), indicating that as control over work pace increases the probability of attending work whilst sick decreases. Criteria set out by Cox and Snell and Nagelkerke [45] indicated that the logistic model explained between 10.7 and 14.4% of the variance in sickness presenteeism.

**Table 4:** Predictors of sickness presenteeism among physicians

Variable	*b*	Wald	OR	95% CI for OR
Unreasonable illegitimate Tasks	0.53*	6.69	1.69	[1.14–2.52]
Role Conflict	0.08	0.33	1.08	[0.83–1.40]
Control over Work Pace	−0.44**	15.48	.64	[0.52–0.80]
Exhaustion	0.59**	11.24	1.81	[1.28–2.55]
Administrative Tasks	0.004	0.55	1.00	[0.99–1.02]
Gender	0.02	0.006	1.02	[0.69–1.50]
Age	−0.07		0.93	[0.85–1.02]
-2log likelihood 619.37** Cox & Snell *R*^*2*^ .107 Nagelkerke *R*^*2*^ .144				

Note. *p < .05, **p < .001

## Discussion

This study has shown that Norwegian hospital physicians have a high unreasonable illegitimate task load, as 50.2% of the participants reported that at least 11% of their task load consisted of unreasonable illegitimate tasks on a daily basis. Approximately 7% of the physicians reported that at least 31% of their daily work tasks were unreasonable illegitimate tasks. Another main finding is that a high unreasonable illegitimate task load was more stressful than a low unreasonable illegitimate task load. Participants with a high unreasonable illegitimate task load reported a higher proportion of administrative tasks and more role conflict than participants with a low unreasonable illegitimate task load. In addition, participants with a high unreasonable illegitimate task load were more likely to attend work whilst sick than colleagues with low unreasonable illegitimate task loads. Moreover, the findings indicate that unreasonable illegitimate tasks and exhaustion both increase the likelihood of sickness presenteeism among physicians, whereas a high control over work pace appears to decrease the odds of physicians attending work whilst sick.

In more detail, this study showed that 50% of all physicians reported that more than 10% of their daily task load consisted of unreasonable illegitimate tasks, i.e. tasks that could have been done by other hospital staff. This finding is consistent with Aronsson and colleagues’ [3] suggestion that physicians spend time on tasks that are not part of their professional role. Our results indicate that a substantial proportion of physicians’ work capacity could be re-allocated to core tasks. On the basis of our results we estimate that between 8 and 13% of daily physician resources could be re-allocated. It follows that re-allocation would increase total use of resources, as employees in other roles would have to compensate for the loss of physician resources to ensure that these tasks continue to be performed effectively, but since physicians can be regarded as a scarce resource, reducing the time they spend on illegitimate tasks should be considered a more efficient allocation of resources.

It seems likely that the tasks physicians define as unreasonable and illegitimate are tasks outside their field of expertise and therefore more appropriate to other (health) personnel. For instance, re-allocating administration of intra-vitreal injections from physicians to nurses was shown to be a resource-efficient usage of personnel that enabled hospitals to cope with an increase in physicians’ workload. DaCosta and colleagues [46] together with Michelotti and colleagues [47] suggested that skill mix (changing professional roles) [48] is beneficial, acceptable to patients, and an effective use of resources. The effectiveness of skill mix across professions remains relatively unexplored [49] and the likely consequences of skill mix change need to be explored [48]. It is important that future research should investigate *the extent to which* skill mix can reduce illegitimate task loads (in a hospital context) or contribute to more efficient use of staff resources. At the same time, future research should identify *which* work tasks physicians consider illegitimate and *why*, as well as exploring *how* such tasks could be made attractive to physicians or other health professionals.

At this point it is important to consider whether the tasks that physicians perceive as illegitimate for a physician really are. For instance, in many professions administrative tasks are increasingly a part of the job description [12]. Administrative tasks might be perceived by physicians as illegitimate because they are not directly related to clinical treatment. We found a positive association between high amount of administrative tasks and unreasonable illegitimate task load amongst physicians after controlling for variance in role conflict. One potential approach to reducing physicians’ perceived amount of unreasonable illegitimate tasks would be to focus on their administrative workload or provide them with the tools they need to manage these tasks more efficiently [19]. As previous research has shown that a high administrative burden causes negative affect and prevents physicians from putting patients first [12], it is important to address and adjust administrative tasks that are perceived as unreasonable and illegitimate. According to the SOS theoretical framework, expecting employees to perform illegitimate tasks is an active violation of the psychological contract between employer and employee, as illegitimate tasks disturb norms and rules associated with a profession [1, 4, 7]. Illegitimate tasks threaten professional identity and are therefore particularly stressful [7]. Thus, being subjected to a high administrative work load and role conflict may make physicians more likely to perceive tasks as illegitimate, because such tasks fall outside their contracted work or professional role. Björk and colleagues [4] found that the following organisational variables were positively associated with managers’ reports of illegitimate task load: internal competition for resources, unfair and illogical resource distribution and poor decisional structure. Our study indicates that physicians have to perform unreasonable illegitimate tasks on an everyday basis, perhaps as a result of poor task distribution within the hospital and those unreasonable illegitimate tasks make it more difficult for physicians to manage their work tasks efficiently Our findings suggest that work tasks other than clinical treatment, research teaching duties that are mandatory for academic physicians are a promising target for interventions aimed at reducing unreasonable illegitimate task load. Further, consistent with earlier findings the current findings suggest that role conflict is related to unreasonable illegitimate task load [7].

This study is consistent with the hypothesis that health is affected by unreasonable illegitimate tasks [4, 7, 10], as it provides evidence that unreasonable illegitimate tasks predict sickness presenteeism amongst physicians when controlling for variance in age, gender, role conflict, control over work pace, exhaustion, and administrative tasks. It is possible that physicians experience unreasonable illegitimate tasks as a kind of attendance pressure that contributes to a conflict between looking after one’s own health and caring for one’s patients. Physicians may feel under pressure to attend work when they are ill because it is patients who suffer when they spend time on unreasonable illegitimate tasks or are absent because of sickness. Another explanation is that physicians attend work whilst ill in order to compensate for high workload [3]. In the long run compensatory sickness presenteeism can be counterproductive and should be avoided, as it could damage the individual, colleges and the organisation [3]. Physicians are known to have high rates of sickness presenteeism [28–30], and our findings suggest that the risk of physicians attending work whilst sick could be reduced by reducing their unreasonable illegitimate task load and level of exhaustion or increasing their control over their work pace.

### Limitations

Although the results provide support for the study hypotheses the cross-sectional design means that we cannot make causal inferences. However, as previous studies have indicated that illegitimate tasks predict strain, it is plausible to suggest that unreasonable illegitimate tasks trigger sickness presenteeism rather than the other way around. The consequences of illegitimate tasks should be investigated through longitudinal research. Common method variance is a potential problem for our study because all the variables were self-reported. But self-reports are probably the most appropriate source of data on illegitimate tasks as the judgement about legitimacy is a matter of individual perception and experience [25]. Generalisability is also an issue, as studies often have single samples of professional/ employees. However analysing data from context-specific samples can be a useful way of developing a systematic understanding of organisational behaviour [50]. This study focused on physicians in academic medicine and we believe that our findings are relevant to work environments and occupations that are similar in terms of unreasonable illegitimate tasks, role conflict and sickness presenteeism.

## Conclusion

Unreasonable illegitimate tasks have only recently been recognised as a workplace stressor and so there has been relatively little systematic investigation of their effects and relationships with other variables [4]. This study contributes to the field by providing evidence on physicians’ experience of unreasonable illegitimate tasks. Overall, our findings provide support for the notion that unreasonable illegitimate tasks should be recognised as a stressor in the field of occupational health psychology, we have identified correlates of unreasonable illegitimate tasks, including sickness presenteeism. Focusing on factors in the work environment should allow us to find ways of reducing unreasonable illegitimate task loads. The study findings indicate that unreasonable illegitimate tasks may leads to potential negative work behaviour such as sickness presenteeism. Although this study does not provide a comprehensive picture of illegitimate tasks or detailed strategies for reducing the proportion of time spent on them, the findings suggest that it would be worth investigating the relationships between unreasonable illegitimate tasks and work attendance and performance. We conclude that physicians spent a high proportion of their work time on unreasonable illegitimate tasks and that strategies should be put in place to reduce this, as performing unreasonable illegitimate tasks may increase sickness presenteeism. Additionally, investing time and effort in the investigating the work environment should make it possible to uncover the antecedents of illegitimate tasks. It seems likely that reducing administrative tasks and role conflict in the workplace would reduce physician’s unreasonable illegitimate task load.

## Abbreviations

ANOVA: Analysis of variance
QPS Nordic: General Nordic Questionnaire for Psychological and Social Factors at Work
SOS: Stress-as-Offense-to-Self (theoretical framework)
the HOUPE study: Health and Organization among University hospital Physicians in Europe
